# Extracellular Vesicles Reflect the Efficacy of Wheatgrass Juice Supplement in Colon Cancer Patients During Adjuvant Chemotherapy

**DOI:** 10.3389/fonc.2020.01659

**Published:** 2020-08-26

**Authors:** Adva Avisar, Miri Cohen, Benjamin Brenner, Tomer Bronshtein, Marcelle Machluf, Gil Bar-Sela, Anat Aharon

**Affiliations:** ^1^The Graduate Studies Authority, University of Haifa, Haifa, Israel; ^2^School of Social Work, Faculty of Social Welfare and Health Sciences, University of Haifa, Haifa, Israel; ^3^Department of Hematology and Bone Marrow Transplantation, Rambam Health Care Campus, Haifa, Israel; ^4^Bruce Rappaport Faculty of Medicine, Technion-Israel Institute of Technology, Haifa, Israel; ^5^The Lab for Cancer Drug Delivery & Cell Based Technologies, The Faculty of Biotechnology and Food Engineering, Technion – Israel Institute of Technology, Haifa, Israel; ^6^Cancer Center, Emek Medical Center, Afula, Israel; ^7^Hematology Research Laboratory, Hematology and Bone Marrow Transplantation, Tel Aviv Sourasky Medical Center, Tel Aviv, Israel; ^8^Faculty of Medicine, Tel Aviv University, Tel Aviv, Israel

**Keywords:** extracellular vesicles, colon cancer, adjuvant chemotherapy, wheatgrass juice, cytokines, thrombogenicity

## Abstract

**Introduction:**

Colorectal cancer (CC) is the third most common type of cancer, accounting for 10% of all cancer cases. Adjuvant chemotherapy is recommended in stages II–III CC. Wheatgrass juice (WGJ) from wheat seeds has high nutritional values, may induce synergistic benefits to chemotherapy and may attenuate chemotherapy-related side effects. Extracellular vesicles (EVs) are subcellular membrane blebs. EVs include exosomes (generated in the endosome, in size <150 nm) and microvesicles (shed from the plasma cell membrane) provide information on their parental cells and play a role in intercellular communication. We aimed to elucidate the effects of chemotherapy administration with supportive treatment of WGJ on CC patients’ EVs characteristics.

**Methods:**

EVs were isolated from the blood samples of 15 healthy controls (HCs) and 50 CC patients post-surgery, treated by chemotherapy, with or without additional daily WGJ. Blood samples were taken before, during, and at the end of chemotherapy. EVs were characterized by size, concentration, membrane antigens and cytokine content using nanoparticle-tracking analysis, western blot, flow cytometry, and protein array methods.

**Results:**

EVs were found to be similar by size and concentration with reduced levels of exosome markers (CD81) on samples at the end of combined treatment (chemotherapy and WGJ). Higher levels of endothelial EVs, which may indicate impairment of the vascular endothelial cells during treatment, were found in CC patients treated by chemotherapy only compared to those with chemotherapy and daily WGJ. Also, EVs thrombogenicity was lower in patients added WGJ compared to patients who had only chemotherapy (levels of tissue factor *p* = 0.029 and endothelial protein C receptor *p* = 0.005). Following treatments, levels of vascular endothelial growth factor receptors (VEGFR-1) and the majority of growth-factors/pro-inflammatory cytokines were higher in EVs of patients treated by chemotherapy only than in EVs obtained from patients with the combined treatment.

**Conclusion:**

Daily consumption of WGJ during chemotherapy may reduce vascular damage and chemotherapy-related thrombogenicity, growth factors and cytokines, as reflected by the characteristics of patient’s EVs.

## Introduction

Colorectal cancer **(CC)** is the third most common type of cancer, accounting for 10% of all cancer cases. Six months of adjuvant chemotherapy with fluoropyrimidines, 5-fluorouracil and leucovorin (5-FU/LV) or capecitabine and oxaliplatin is the current worldwide standard of care for patients with stage III colon cancer following curative surgery (1). Patients with low-risk stage II disease can be followed without adjuvant therapy, or considered for adjuvant treatment with capecitabine or 5-FU/LV. For patients with high-risk stage II disease, adjuvant therapy with capecitabine or 5-FU/LV can be considered, as for treatment of stage III disease, according to numerous risk factors (2). Adjuvant chemotherapy affects many aspects of quality of life and there is an urgent need to mitigate them. A wide range of health benefits has been attributed to wheatgrass juice (WGJ), the young grass of the common wheat plant *Triticum aestivum*. Its components include chlorophyll, flavonoids, 17 amino acids, eight of which are essential, vitamins A, C, and E, and high mineral content (iron, calcium, magnesium, zinc) (3). *In vitro* studies, mostly using the fermented wheat germ extract, have demonstrated anti-cancer potential and have identified apoptosis induction, and anti-proliferative and anti-angiogenic effects as a possible mechanism (4). In animal experiments, wheatgrass demonstrated benefits in cancer prevention and as an adjunct to cancer treatment, as well as benefits to immunological activity and oxidative stress (3). Clinical trials show that wheatgrass may induce synergistic benefits to chemotherapy and may attenuate chemotherapy-related side effects (3). For example, in a study of 6-month supplementation of fermented wheat germ extract to anticancer treatments of CC, lower rates of recurrence and longer progression-free and overall survival were reported (5).

The effect of frozen WGJ on myelotoxicity induced by chemotherapy was assessed among chemotherapy-naïve breast cancer patients (6). In this matched pairs study, patients received WGJ daily during the first three cycles of chemotherapy, while matched patients received only chemotherapy. The most important effect observed was a reduction in neutropenic fever events and in neutropenic infections. However, all clinical studies were missing any basic explanation of the reported results. Extracellular vesicles (EVs) may reflect body response to distress and may facilitate the understanding of influences of nutritional support during chemotherapy. EVs, including exosomes (produced in the endosomal compartment in size <150 nm) and microvesicles (shed from the plasma cell membrane, in size <1 micron) play a role in intercellular communication within the tumor microenvironment (7). As such, EVs modulate target cells by delivering oligonucleotides and proteins, for example the (EGFR vIII) that is provided by glioma EVs to cancer cells lacking this receptor (8). In addition, EVs carry numerous growth factors (9) which can be transferred to recipient cells and genetic material (DNA, RNA, microRNA) that alter cell signaling pathways (10). Thus, EVs can affect tumor cells and their related microenvironment function, leading to cells differentiation, proliferation, migration, invasion, and overall tumor progression. More specifically, it has been shown that colon cancer EVs are enriched with specific proteins (11) and cell cycle-related mRNAs that promote endothelial cells proliferation (12). Thus, EVs can also modulate the immune activity of monocyte-derived macrophages and thereby reduce the tumor restrictive capacity (13). EVs obtained from colon cancer patients in stage II/III/IV were found to be more thrombogenic compared to EVs obtained from healthy controls (HCs) (14). In a previous study on breast cancer patients during adjuvant chemotherapy, we demonstrated that tumor EVs are present in the plasma for long periods after tumor removal (15). In recent years, as a result of their properties, EVs from defined cell types were proposed as novel biomarkers which reflect disease severity and treatment efficacy, and may provide novel diagnostic information. Therefore, in order to define a biomarker for treatment efficacy, we aimed to elucidate the effects of chemotherapy administration, with or without supportive treatment of wheatgrass therapy, on the characteristics of EVs obtained from CC patients.

## Materials and Methods

### Study Population

Patients for the current study were recruited between March 06, 2014 and May 10, 2016. The study was approved by the Institutional Review Board of Rambam Health Care Campus (RHHC) in Haifa, Israel (Approval No. 0153-13). The selection of suitable candidates for the study was conducted through the hospital’s computerized system.

Inclusion criteria: Patients diagnosed with colon cancer at stages II–III toward adjuvant chemotherapy following curative surgery. Each patient found to be suitable according to these criteria was asked to participate in the study. Letters were sent to suitable candidates to inform them of research being conducted in the Division of Oncology at RHHC. A week later, the patients were telephoned to suggest participation in the study, as well as to provide information and explanation regarding the study and to answer questions. Patients who were interested in participating were admitted to the WGJ (CC-W) group. Patients who preferred not to participate were asked to donate blood samples for the purpose of the study, and those who agreed were entered into the standard treatment (CC-C) group. All participants signed an informed consent form prior to their participation.

The study population included 15 HC and 50 CC patients, aged 18 plus, at stages II–III who received adjuvant chemotherapy following curative surgery at RHHC. Patients were divided into two subgroups: the CC-C group included 25 patients treated by standard chemotherapy (1. capecitabine alone or 2. capecitabine + oxaliplatin or 3. 5-FU/LV + oxaliplatin); and the CC-W group that included 25 patients who received a combination of the same standard chemotherapy with support treatment of WGJ daily. The patients in the CC-W group were given monthly supplements of frozen WGJ 60 cc packages and were asked to take one package of 60 cc daily, in the morning, on an empty stomach. Adherence to these instructions during the study period was 80%.

Wheatgrass juice is squeezed from the organic mature sprouts of wheat seeds (*T. aestivum*). For product quality control (storage and transport) frozen WGJ were used in the current study, supplied by a single agriculturist. The wheatgrass was grown on unified compost, consisting entirely of organic manure and organic materials only. Wheat seeds were germinated on trays that contained the compost throughout the year. There was an automatic constant irrigation during the growth. Harvest took place whenever the wheat sprouts reached a length of about 15–20 cm (when their nutritional values are at peak). The content of nutritional components of the wheatgrass grown in this method does not change throughout the year (although the sugar content is slightly higher on sunny days compared to cloudy days). Squeezing was always cold press – done by a special slow rpm juicer. As a result, the grass and the juice stays cool throughout the process, and nutritional values are preserved. Also, squeezing was carried out immediately before freezing, in order to maintain nutritional values.

**FIGURE 1:**
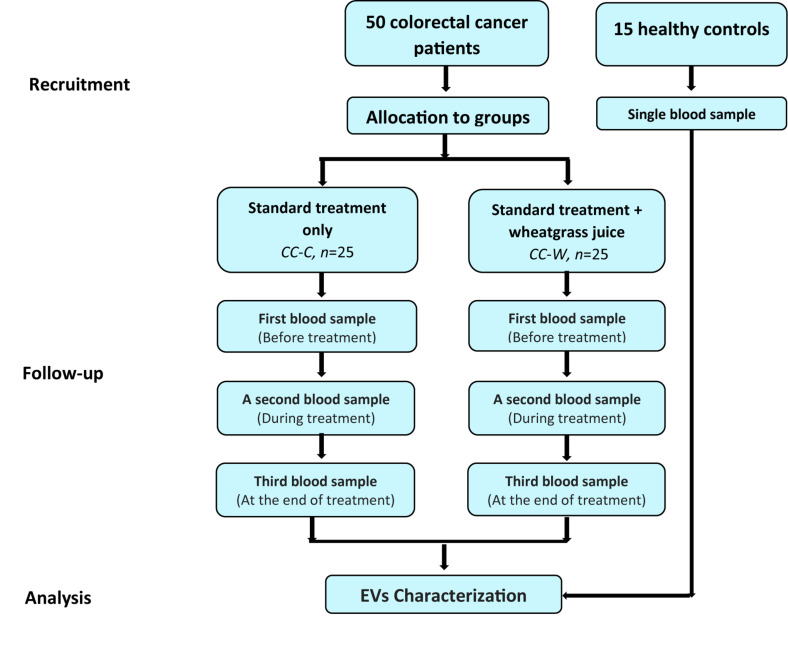
Study design flow chart. Patients diagnosed with colon cancer at stages II–III toward adjuvant chemotherapy following curative surgery. Patients who were interested in participating were admitted to the WGJ (CC-W) group. Blood samples were collected from CC-W and from non-WGJ patients, following consent. Blood samples were obtained at three time points: before first chemotherapy (time point I – BT), after 3 months of chemotherapy (time point 2), and at the end of chemotherapy administration (time point 3). One blood sample was collected from each healthy control (HC). PPP EVs were analyzed.

After signing a consent form, blood samples were obtained from each patient at three time points: before the first chemotherapy (time point I – before treatment (BT), after 3 months of chemotherapy (time point II), and at the end of the chemotherapy administration (time point III). One blood sample was collected from each one of the HC. The study design is described in Figure 1.

### EVs Isolation

The aim of the current study was to characterize total circulating EVs. This study does not intend to separate between the large and small vesicles populations (MVs and exosomes). EVs were isolated as described previously (15, 16). Briefly, 15 ml of peripheral venous blood were drawn from study participants into sodium citrate (3.2%) tubes; platelet-poor plasma (PPP) was obtained after two centrifugations (15 min, 1500 *g*) within an hour of collection and frozen at −80°C. EVs size, concentration, and membrane antigens levels were validated on PPP samples. EVs pellets were isolated from thawed PPP by centrifugation (1 h, 20,000 *g*, 4°C). Liquid supernatants were discarded, and EV pellets were used for additional analysis by western blot (WB) and protein array.

### EVs Characterization

#### EVs Size and Concentration

Plasma EVs size and concentration were evaluated by nanoparticle-tracking analysis (NTA) that can measure particles in the range of 50–2000 nm (17). NTA was performed using a NanoSight NS300 system with a CMOS camera and 532-nm laser (Malvern Instruments, Malvern, United Kingdom). Each sample was measured three times.

### Exosome Marker

Exosome membrane is highly enriched with Tetraspanins (CD63, CD81), suggested as exosome markers (18); therefore, EVs obtained from equal amounts of plasma (300 μl) of study groups were loaded on 12% acrylamide gel and transferred to immune blot-PVDF membrane (Bio-Rad, Hercules, CA, Untied States). The blots were probed with mouse monoclonal anti human-CD81 or CD63 (Santa Cruz Biotechnology, Santa Cruz, CA, United States), followed by staining with peroxidase-conjugated secondary antibody goat anti mouse IgG HRP (Santa Cruz, CA, United States). The blots were developed with chemiluminescent substrate reagents (EZ-ECL, Biological Industrial, Israel) and scanned by MY-ECL Imager (Thermo Fisher Scientific, Waltham, MA, United States); the data of four experiments were summarized as percentages of HC.

### Membrane Antigens

Antigen levels of EVs were evaluated by flow cytometry (FACS-CyAn ADP analyzer, Beckman Coulter). EVs were labeled with fluorescein isothiocyanate (FITC)-Annexin V (Bender MedSystems, Vienna, Austria) that binds to negatively charged phospholipids and with specific fluorescent antibodies: FITC-CD235 [red blood cells (RBC) marker], allophycocyanin (APC)-Flt-1 (vascular endothelial growth factor receptor (VEGF-R-1), APC IgG1κ isotype control and anti-epithelial tumor antigen A33-alexa flour 488 (R&D Systems, Minneapolis, MN, United States), FITC-CD14 (monocytes, IQ Products, Netherlands), PE-CD11a (leukocytes). FITC-CD31 (endothelial cells), FITC IgG isotype controls, PE-IgG1k isotype control and APC IgG isotype control (BD Biosciences, San Jose, CA, United States) Phycoerythrin (PE)-CD41 (platelet marker), PE-CD62P (activated platelet marker), PE-anti-CD62E (E-selectin), APC-CD144 (Bio-Legend, CA, United States). Coagulation markers: FITC anti-human tissue factor (TF) and anti-human tissue factor pathway inhibitor (TFPI) (America Diagnostica, Greenwich, CT, United States) anti-mouse IgG-PE (Jackson, PA, United States). PE-Thrombomodulin and PE were purchased from BD Pharmingen (San Jose, CA, United States), FITC anti-human endothelial protein C receptor (EPCR) from Santa Cruse, Dallas, TX, United States.

Pro-coagulant activity was evaluated using the Factor X activated (FXa) assay as previously described (19). Briefly, 25 μl of EV pellet isolated from 1 ml PPP, were mixed in 50 μl of Ca^+2^ Tris buffer (25 mM, pH 8) with 25 μl FVIIa (Novoseven, Novo Nordisk, Copenhagen, Denmark) and 1 ng/ml FX (American Diagnostica), and incubated for 15 min at 37°C. Then, chromogenic substrate was added (2.5 mM Spectrozyme FXa, American Diagnostica, Greenwich, CT, United States) for an additional incubation time of 30 min at 37°C. Samples’ absorbance at 405 nm was then measured with a plate reader.

Extracellular vesicles protein content was screened by the Human Angiogenesis Protein Antibody Array (Ray Bio, Norcross, GA, United States). EV protein extract was obtained from a pool of five specimens within each patient sub-group and quantified using the bicinchoninic acid (BCA) protein quantification kit (Thermo Fisher Scientific Inc., IL, United States). Each protein array slide was loaded with 90 μg of protein from the EVs pool lysate and the array was performed according to the manufacturer’s instructions. Expression of each protein was represented in quadruplicate on the array slides. Quadruplicate dots identifying each protein were scanned and quantified by GenePIx 4000B (Molecular Devices, Sunnyvale, CA, United States). The mean fluorescence intensity of these dots (AU) was determined for intergroup comparisons.

### Statistical Analysis

Data were analyzed using SPSS (IBM SPSS statistics version 25, IBM Crop Armonk, NY, United States, 2017). Pearson Chi-Square or Exact Fisher test were used to study exact significance between groups and the Mann–Whitney U non-parametric test was used to assess significant differences of continuous or ordinal dependent variables by a single dichotomous independent variable test. The generalized estimating model was used to evaluate change during the follow-up in each group and to test if the change in the groups during follow-up was significantly different (*p* < 0.05 was considered as significant). All statistical tests were 2-sided. An increase of >50% or a decrease by >30% were considered as significant changes. MedCalc statistical software, Comparison of proportions calculator were used.

## Results

### Study Population

Blood samples were collected from 15 HC (age 62.9 ± 7.3 years) and from 50 CC patients (age 60.6 ± 11.42 years) post-surgery, who received adjuvant treatment. The majority of HC were women (67%) while the majority of patients were males (72%, *p* = 0.0034). The patients group was sub-divided into two sub-groups: the CC-C group included 25 patients treated by standard chemotherapy: capecitabine (12%), capecitabine + oxaliplatin (72%) or folinic acid, 5FU + oxaliplatin (16%), while the CC-W group included 25 patients (age 65.0 ± 8.3 years, 84% male) who received capecitabine (16%), capecitabine + oxaliplatin (80%) or folinic acid, 5FU + oxaliplatin (4%) with support treatment of 60 cc WGJ daily. Patient characteristics are summarized in Table 1. Although allocation in the study was not random, no significant demographic or clinical differences were observed between the WGJ (CC-W) group and the standard treatment (CC-C) group (Table 1). However, significant changes along the study period were found in WBC count (reduction) in the CC group (*p* = 0.001) and in monocyte count (increase) in the CC-W group (*p* = 0.002). Patients in the current study were recruited consecutively; 88% were in stage III colon cancer leading to including oxaliplatin in the chemotherapy regimen in 85% of the entire group (37 in combination with capecitabine and 4 with 5-FU). Capecitabine was given to 90% of the patients (7 as the only drug). EVs obtained from patient subgroups of chemotherapeutic type regimens were analyzed first according to the type of regimen. There were no statistically significant differences in the characteristics of EVs among the different chemotherapy groups. Hence, we united the subgroups of chemotherapy into one consolidated group and analyzed the EVs data according to the intervention of WGJ consumption. Small differences in the EV characteristics in the groups without oxaliplatin or with continued 5-FU instead of capecitabine were unable to be identified.

**TABLE 1:** Patient characteristics.

	Healthy controls (HC) *N* = 15	Patients (all) *N* = 50	Patients: standard treatment only *N* = 25	Patients: standard treatment + wheatgrass juice (WGJ) *N* = 25	Statistics (*P*) Fisher’s exact test
Age, years					
Median (min–max)	65 (49–72)	63 (29–83)	66 (45–83)	63 (29–75)	
Males, *n* (%)	5 (33.3%)	39 (78%)	18 (72%)	21 (84%)	$ = 0.003
Females, *n* (%)	10 (66.7%)	11 (22%)	7 (28%)	4 (16%)	$ = 0.003
Cancer disease:					
Stage II	–	6 (12)	2 (8%)	4 (16%)	
Stage III	–	44 (88)	23 (92%)	21 (84%)	
**Chemotherapy:**					
*n* (%) capecitabine	–	7 (14.0)	3 (12%)	4 (16%)	
Apecitabine, oxaliplatin	–	38 (76.0)	18 (72%)	20 (80%)	
5-fluorouracil, leucovorin, oxaliplatin	–	5 (10.0)	4 (16%)	1 (4%)	
**Other parameters:**					
Heart disease, *n* (%)	1 (6.7)	2 (4.0)	1 (4)	1 (4)	
Diabetes, *n* (%)	2 (13.3)	8 (16.0)	6 (24)	2 (8)	
Hypertension, *n* (%)	1 (6.7)	18 (36.0)	9 (36)	9 (36)	
Hyperlipidemia, *n* (%)	2 (13.3)	6 (12.0)	3 (12)	3 (12)	
Anticoagulants, *n* (%)	0	13 (26.0)	7 (28)	6 (24)	

*$-patient vs. HC.*

### EVs Characterization

**FIGURE 2:**
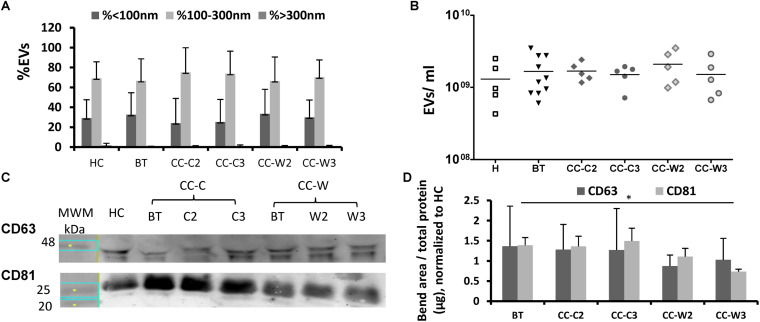
EVs size distribution, concentration and exosome markers. EVs were obtained from the study groups: healthy controls (HC) and colon cancer patients during adjuvant chemotherapy only CC-C or adjuvant chemotherapy with WGJ (CC-W). Samples obtained before treatment (BT, time point 1), during treatment (time point 2) and at the end of treatment (time point 3). EVs were isolated by a series of centrifugations Nanoparticle-tracking analysis-determined EV size distribution **(A)** and concentration **(B)**. *N* = 5 for each study group. WB analysis with antibodies against exosome markers CD63 and CD81. Representative gel with molecular weight marker (MWM) and representative sample of each study cohort **(C)** graph summaries the bends area divided with protein concentration of each sample that were loaded to the gel. *N* = 3 **(D)**.

Extracellular vesicles size, concentration and membrane antigens were validated on the total EVs population as they appear in PPP without additional isolation steps. Similar EVs average size (∼140 nm) and concentration (∼10^9^ EVs/ml) were found in the study cohorts and the majority of EVs (∼70%) were 100–300 nm in size (Figures 2A,B). However, WB analysis performed on EVs pellets obtained by 20,000*g* centrifugation for 1 h displayed reduced levels of exosome markers (CD81) at the CC-W3 sample compared to EVs BT (*p* = 0.016) (Figures 2C,D).

### EVs Cell Origin

**FIGURE 3:**
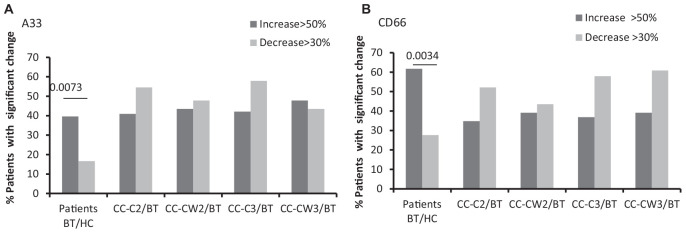
EVs tumor cell markers. Antigen levels of tumorigenic markers were measured on EVs obtained from healthy controls and on EVs obtained from patients (CC-C and CC-W) before chemotherapy (BT, time point 1), during treatment (time point 2), and at the end of chemotherapy treatment (time point 3). The percentage of labeled EVs was calculated from the total number of EVs using FACS analysis. The graph presents significant changes (>50% increase or >30% decrease) in patient EVs levels BT compared to HC and the change during treatment and at the end of chemotherapy treatment compared to the levels BT of EVSA33 antigen **(A)**; EVs CD66 **(B)**.

Significantly higher levels of the CC markers, the carcinoembryonic antigen (CD66), and a trend of increase of the A33 protein were found compared to HC. Specifically, compared to the average of HCs, more CC patients express higher levels of the cancer markers BT (CD66-EVs 59% of patients; A33-EVs 40% of patients) while only a minority of CC patients express lower levels of the cancer markers BT (CD66-EVs 28% of patients; A33-EVs 16.7% of patients) (Figure 3 and Table 2). No significant differences were found in the expression of CD66- and A33 between patient’s subgroups EVs. Levels of RBC EVs, expressed CD235a, and Glycophorin A were significantly higher in patients who received chemotherapy only (CC-C) than in CC-W patients, indicating high RBC damage related to this group. RBC-EVs obtained from HC and patients treated with WGJ (CC-W) displayed similar levels (Table 2). However, because EVs obtained BT from CC-W patients displayed lower levels of the RBC-marker than CC-C patients, the addition of WGJ to the treatment cannot be considered as an effector on RBC EVs levels.

**TABLE 2:** EV cell origin membrane antigen characteristics.

EVs markers	HC	CC-BT	CC-C2	CC-C3	CC-W2	CC-W3	Statistics
**Cell origin antigens**							Mann–Whitney U; Wilcoxon W
**CD66**							
*n*	11	40	18	15	19	17	HC vs. BT, 0.018 HC vs. CC-C2, 0.001 HC vs. CC-C3, 0.001 HC vs. CCW2, 0.018 HC vs. CCW3, 0.013
Mean	3.19	9.87	13.17	11.87	15.99	8.4	
Median	1.1	9.05	9.35	11.4	6	6.2	
Interquartile range (IQR)	0.6–14.1	3.07–16.05	6.82–17.78	5.2–33.4	1.6–26.7	3.05–25.2	
**A33**							
*n*	13	40	22	18	23	25	HC vs. CC-C2, 0.007 HC vs. CC-C3, 0.043
Mean	6.16	16.65	16.21	17.61	15.86	12.22	
Median	3.40	8.05	12.70	12.20	5.500	7.20	
IQR	1.85–11.15	3.90–34.53	7.40–20.10	6.37–26.80	1.60–25.40	2.10–21.35	
**RBC (CD235a)**							
*n*	15	49	23	20	25	25	HC vs. CC-C2, 0.026 CC-C3 vs. CC-W3, 0.008 CC-C3 vs. CC-W3, 0.036
Mean	33.44	45.78	53.22	47.68	35.11	36.61	
Median	26.50	45.60	57.80	48.55	28.90	32.10	
IQR	10.9–50.30	34.20–59.15	43.50–69.70	37.95–64.63	16.90–48.85	21.95–45.85	
**Platelet (CD41)**							
*n*	11	39	18	14	19	23	HC vs. BT, 0.045 HC vs. CC-W2, 0.007 HC vs. CC-W3, 0.003 CC-C3 vs. CC-W3, 0.004
Mean	16.78	5.25	9.78	11.34	4.74	4.26	
Median	11.20	3.10	7.95	8.85	4.50	3.10	
IQR	4.80–23.40	0.80–7.200	3.85–13.85	4.52–13.85	1.80–7.100	0.20–6.70	
**Activated platelet (CD62P)**							
*n*	15	50	23	20	25	25	HC vs. CC-W2, 0.014 HC vs. CC-W3, 0.043
Mean	4.37	1.67	2.93	3.09	0.81	1.33	
Median	4.40	0.05	0.70	1.15	0.0	0.0	
IQR	0.0–5.40	0.0–1.60	0.0–4.00	0.0–4.10	0.0–1.35	0.0–1.05	
**Leukocyte (CD11a)**							
*n*	13	39	23	19	25	25	
Mean	7.41	4.34	5.32	7.66	4.45	4.94	
Median	4.10	2.90	4.30	6.90	4.30	4.70	
IQR	1.85–9.00	0.0–7.15	2.50–7.00	1.30–8.30	1.20–6.60	3.05–7.60	
**Monocyte (CD14)**							
*n*	14	49	23	19	25	25	HC vs. BT 0.05 HC vs. CC-C2, 0.046 HC vs. CC-W2, 0.03 HC vs. CC-W3, 0.016
Mean	8.42	3.77	4.52	7.02	4.09	3.64	
Median	8.30	1.50	3.60	4.60	2.80	2.40	
IQR	2.77–13.60	0.0–5.10	0.0–9.20	0.60–8.60	0.0–6.75	0.55–5.10	
**CD144**							
*n*	13	49	23	19	25	25	HC vs. CC2, 0.02 HC vs. CC3, 0.018 CC-C3 vs. CC-W3, 0.035
Mean	33.28	43.15	48.79	50.33	43.69	38.24	
Median	29.30	38.30	52.40	52.00	40.60	38.20	
IQR	23.10–47.75	26.85–62.35	32.20–61.40	32.00–63.20	26.55–68.05	21.55–48.80	
**CD31+/41−**							
*n*	13	50	23	20	25	25	HC vs. CC-C2, 0.02 HC vs. CC-C3, 0.001 CC-C2 ver. CC-W2, 0.039; CC-C3 ver. CC-W3, 0.002
Mean	4.33	9.39	12.78	12.77	8.98	6.344	
Median	3.40	5.55	11.70	13.45	4.80	3.800	
IQR	2.15–6.60	3.27–11.45	4.80–19.50	5.60–18.65	2.70–11.60	1.750–8.600	

The expression of platelet and activated platelet markers (CD41 and CD62P, respectively) was found to be significantly lower in EVs obtained from all patients BT compared with HC, with an additional reduction in CD41 measured in patients during treatment with WGJ compared to those treated with chemotherapy alone (Table 2).

White blood cells EVs, such as leukocyte derived EVs, labeled with CD11, were found to be similar in both patient sub-groups, while lower levels of monocyte, CD14-expressing EVs were found in patients BT than in HC and during treatment in the treated groups (Table 2).

**FIGURE 4:**
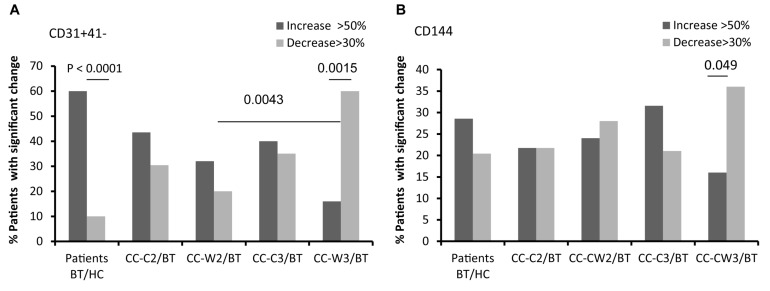
EVs endothelial cell markers. Antigen levels of endothelial markers were measured on EVs obtained from healthy controls and on EVs obtained from patients (CC-C and CC-W) before chemotherapy (BT, time point 1), during treatment (time point 2), and at the end of chemotherapy treatment (time point 3). The percentage of labeled EVs was calculated from the total number of EVs using FACS analysis. The graph presents significant changes (>50% increase or >30% decrease) in patient EVs levels BT compared to HC and the change during treatment and at the end of chemotherapy treatment compared to the levels BT of EVs CD31 + /CD41 **(A)**; EVs CD144 **(B)**.

The levels of endothelial cells EVs (EC-EVs, CD31^+^/CD41^–^) obtained from both patient sub-groups BT were found to be twice as high as those obtained from HC, Specifically, 60% of patients BT demonstrated higher levels of EC-EV (EVs CD31+/CD41−) than HC, while only 10% of the patients BT have lower levels of EVs CD31+/CD41− (*p* < 0.0001) (Figure 4A and Table 2). The level of EC-EVs remained high during standard chemotherapy treatment, and it was significantly decreased in the group treated with a combination of chemotherapy and wheat juice (CC-C2: vs. CC-W2 (*p* = 0.039); CC-C3 vs. CC-W3 (*p* = 0.002). Specifically, at the end of combined treatment, 60% of patients demonstrated a decrease in EC-EVs (CD31+/CD41−) but only 16% of these patients demonstrated an increase in EVs CD31+/CD41− (*p* = 0.0015). In contrast to the combined treatment, only 20% of patients with standard chemotherapy displayed a reduction of EVs CD31+/CD41− (*p* = (0.0043at the end of treatment.

Similar differences between patient subgroups were found in other endothelial markers. The levels of CD144-EVs (Figure 4B and Table 2) were higher in the CC-C3 group during and at the end of standard treatment than in HC and significantly lower in the group treated with the addition of WGJ (CC-C3 vs. CC-W3, *p* = 0.035). Specifically, 36% of patients at the end of combined were demonstrate significant decrease in EVs expression of CD144 while only 16% of patients from this group presented increase in CD144-EVs (*p* = 0.049).

### EVs Thrombogenicity

**FIGURE 5:**
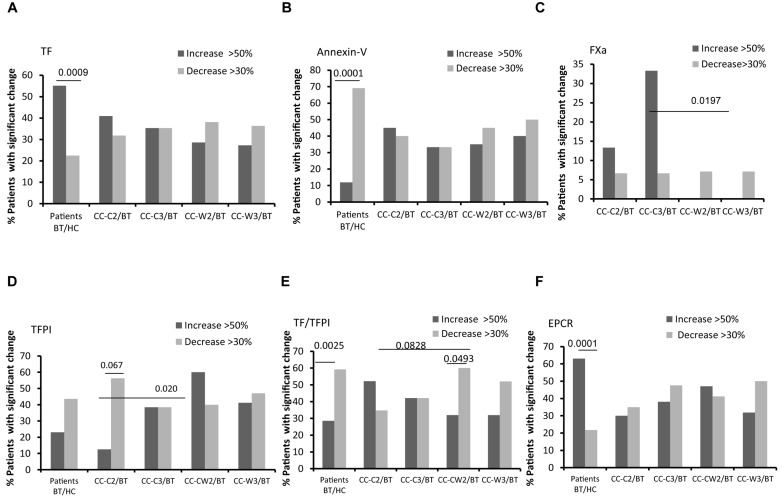
EVs thrombogenicity. Antigen levels of coagulation markers were measured on EVs obtained from healthy controls and from patients before chemotherapy (BT, time point 1), during treatment (time point 2), and at the end of chemotherapy treatment (time point 3), using specific fluorescent antibodies. The percentage of labeled EVs was calculated from the total number of EVs using FACS analysis. The graph presents significant changes (>50% increase or >30% decrease) in patient EVs levels BT compared to HC and the change during treatment and at the end of chemotherapy treatment compared to the levels BT related to% TF labeled EVs **(A)**; % TFPI labeled EVs **(B)**; TF/TFPI EVs ratio **(C)**; % EPCR labeled EVs **(D)**; % Annexin V **(E)**; % EVs procoagulant activity was measured by factor X chromogenic assay **(F)**.

Levels of EVs expressing TF, the coagulation activator, were twice as high in patients BT compared to HC (*p* = 0.05). Specifically, 55.1% of patients expressed significant higher levels of EV-TF compared to HC. While the TF-expressing EVs level remained stable during treatment with chemotherapy combined with WGJ (with trend of decrease), it increased along the standard chemotherapy period with significant differences found between the treatments (CC-C2 vs. CC-W2, *p* = 0.039 and CC-C3 vs. CC-W3, *p* = 0.002) (Figure 5A and Table 3).

**TABLE 3:** EV coagulation factors.

	HC	CC-BT	CC-C2	CC-C3	CC-W2	CC-W3	Statistics
**Coagulation markers**							Mann–Whitney U; Wilcoxon W
**Annexin V**							
*n*	11	36	19	15	15	17	HC vs. CCW3, 0.016 CC-C3 vs. CC-W3, 0.052
Mean	13.9	8.21	12	9.33	6.5	4.57	
Median	9.7	4.95	6.1	7.9	4.9	3.7	
IQR	4.4–26.2	1.47–11.3	2.3–17.5	3.6–12.7	3.6–6.8	2.05–5.86	
**TF**							
*n*	15	48	22	18	21	22	HC vs. BT 0.05 HC vs. CC-C2, 0.015 HC vs. CC-C3, 0.006 CC-C2 vs. CC-W2, 0.065 CC-C3 vs. CC-W3, 0.029
Mean	7.7	14.4	20.35	20.05	11.79	10.33	
Median	7.1	12.1	15.3	15.75	9.3	6.4	
IQR	3.4–9.2	6.3–31.5	6.2–32.27	8.65–29.97	2.5–16.85	4.15–15.5	
**TFPI**							
*n*	15	45	22	18	21	22	CC-C2 vs. CC-W2, 0.044
Mean	6.26	5.85	4.3	4.2	9.11	7.05	
Median	5.6	4.6	2.6	2.4	7.1	4.4	
IQR	1.84–7.5	2.05–8.8	1.05–4.97	0.5–7.02	2.05–11.1	1.15–10.7	
**TF/TFPI ratio**							
*n*	15	45	22	18	21	22	CC-C2 vs. CC-W2, 0.008
Mean	4.71	6.5	10.3	9.06	3.2	4.1	
Median	1.43	2.79	4.4	3.96	1	1.68	
IQR	0.8–7.17	1.02–11.3	1.22–15.4	1.45–8.75	0.39–4.6	0.5–6.3	
**EPCR**							
*n*	14	46	20	18	24	24	HC vs. BT 0.01 HC vs. CC-C2, 0.002 HC vs. CC-C3, 0.000 CC-C2 vs. CC-W2, 0.063 CC-C3 vs. CC-W3, 0.050
Mean	3.76	15.01	18.8	13.37	11.5	10.05	
Median	2.75	8.85	13.1	10.95	3.7	5.7	
IQR	1.6–6.3	3.1–19.7	4.77–25.57	5.7–10.02	1.5–14.02	1.67–11.3	
**Thrombomodulin**							
*n*	15	49	23	19	25	25	
Mean	3.76	6.29	7.5	7.64	4.44	4.3	
Median	2.75	1.2	4.1	3.2	1.4	2.5	
IQR	1.62–6.32	0–6.7	0.2–6.9	0.2–7.9	0.15–2.25	1.15–4.65	

Levels of EVs expressing the anticoagulant tissue factor pathway inhibitor (TFPI) were found to be similar in HC and in the patient’s cohort BT; however, this level significantly increased in the CC-W2 patients compared to CC-C2 patients (*p* = 0.014) (Table 3). The majority of patients with combined treatment demonstrated an increase in TFPI EVs during treatment (60%), while only 18% of patients with standard treatment demonstrated a similar trend (*p* = 0.0204) (Figure 5B). Overall, the ratio between TF and its inhibitor (TF/TFPI ratio) on EVs were found to be significantly higher on EVs obtained from patients during standard treatment than EVs obtained from patients treated with WGJ as well (*p* = 0.008, Table 3). Moreover, the majority of patients with combined treatment presented decrease in EVs TF/TFPI during and at the end of treatment (CC-W2 60% and CC-W3 50% of patients) compared to their state BT (Figure 5C). In addition, an increase was detected in the EV levels of EPCR in patients BT compared to HC (*p* = *0.01*). Specifically, 63% of patients BT express significant higher levels of EV-EPCR while only 21% of patients BT present reduced levels of EVs-EPCR (*p* < 0.0001) compared to HC. The level of EPCR-EVs remained constant during standard chemotherapy treatments and decreased in EVs obtained from combined treatments with WGJ (CC-C2 compared to CC-W2, *p* = *0.0*63; CC-C3 compared to CC-W3, *p* = 0.05) (Figure 5D and Table 3). However, levels of thrombomoduline (TM) bearing EVs remained constant in the study groups (Table 3). Reduced EVs’ labeling by Annexin V were found in the majority of CC patients BT compared to an average of HC EVs (69% of patients, *p* < 0.0001) (Figure 5E).

Coagulation activity assays as measured by FXa chromogenic assay demonstrated non-significant changes in EVs’ thrombogenic activity between groups, However, more patients of the CC-C group presented a significant increase in EVs procoagulant activity (>50% increase) during and at the end of treatment (13 and 33% of patients), while no significant increase in procoagulant activity was documented in any patient with the combined treatment during and at the end of treatment (*p* = 0.1647 and *p* = 0.0197), respectively (Figure 5F).

### EVs Cytokine Content and Expression of Growth Factor Receptors

**FIGURE 6:**
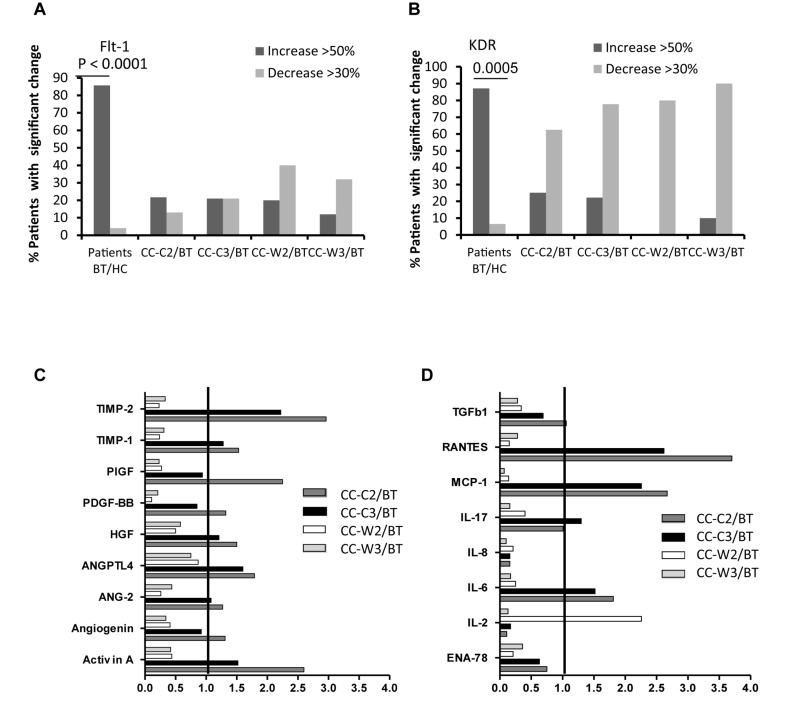
EVs expression of VEGF-receptors (Flt-1, KDR) and cytokine contents. Levels of growth factors receptors were measured on EVs obtained from healthy controls and on EVs obtained from patients at three time points, using specific fluorescent antibodies; The graph presents significant changes (>50% increase or >30% decrease) in patient EVs levels BT compared to HC and the change during treatment and at the end of chemotherapy treatment compared to the levels BT related to **A**. VEGFR1 (Flt-1) **(A)**, VEGFR2 (KDR) **(B)**. EV proteins extract, obtained from a pool of five specimens within each patient subgroup, were validated by Human Angiogenesis Protein Antibody Array. The graph presented the change in specific EVs cytokine and growth factors content during treatment compared the EVs content before treatment. Growth factors **(C)** and inflammatory cytokine **(D)**.

The levels of EVs VEGFR1 (Flt-1) were found to be significantly higher in all CC patients BT compared to EVs obtained from HC (HC 15.48 ± 14.77% vs. BT 42.87 ± 21.97, *p* = 0.0002) specifically, 85.7% of patients expressed significant higher levels of EV-Flt-1 compared to HC (Figure 6A). However, significantly reduced levels of Flt1expresing EVs were found in patients who received combined treatment of chemotherapy and WGJ compared to the standard chemotherapy group, as measured during and at the end of the treatment course (CC-C2: 53.08 ± 16.27% vs. CC-W2: 36.62 ± 36.62%, *p* = 0.005; CC-C3: 47.50 ± 18.10% vs. CC-W3: 33.59 ± 15.57%, *p* = 0.0110) (Table 4). Overall, the mean change in EVs VEGFR1 between time points was found to be significant only in the CC-W group (*p* = 0.008).

**TABLE 4:** EVs growth factors receptors.

	HC	CC-BT	CC-C2	CC-C3	CC-W2	CC-W3	Statistics
**Growth factors receptors**							Mann–Whitney U; Wilcoxon W
**VEGF-R1 (Flt-1)**							
*n*	14	49	23	19	25	25	HC vs. BT, 0.0000 HC vs. CC-C2, 0.000 HC vs. CC-C3, 0.0004 HC vs. CCW2, 0.005 HC vs. CCW3, 0.007 CC-C2 vs. CC-W2, 0.011 CC-C3 vs. CC-W3, 0.018
Mean	16.74	43.62	49.61	45.09	35.60	33.21	
Median	13.00	43.60	49.80	44.60	33.10	27.70	
IQR	3.55–29.93	28.15–57.15	37.90–69.30	26.90–63.30	24.75–43.90	22.05–45.90	
**VEGF-R2 (KDR)**							
*n*	9	32	8	9	10	12	HC vs. BT, 0.001 HC vs. CC-C3, 0.014
Mean	7.8	26.54	15.8	20.3	15.9	12	
Median	5	27.65	14.6	13.5	12.4	12.7	
IQR	3.8–9.85	14.5–33.5	5.4–25	6.85–30.8	5.6–28.6	4.9–16.37	
**Tie-1**							
*n*	3	13	6	5	6	6	
Mean	26.87	30.18	32.58	37.9	23.3	21.9	
Median	17	33.3	34.45	38.9	25.4	19.4	
IQR	8.6–55	26.75–34.75	25.35–41.25	20.8–54.5	8.8–35.95	15.05–28.42	

The majority of the patients (87%) expressed also significant higher levels of EV-VEGFR2 (KDR) compared to HC (Figure 6B). A significant reduction was found in the expression EV-KDR during and at the end of treatment in the combined treatment with WGJ compared to the CC-C group (CC-W2, *p* = 0.008, and CC-W3 *p* = 0.0001) (Table 4). Trend of increase in the expression of TIE1, the angiopoietin receptors was found in the patients group who received chemotherapy only (*p* = 0.012) (Table 4).

A protein array was performed on patients’ EVs pellets and also on the supernatant that was discarded after centrifuging of 20,000*g* for 1 h and used to screen for the content of 60 cytokines and growth factors in EVs compared to their free forms in the plasma. For most proteins, their level in the EVs was found to be equal or greater than in the plasma (Supplementary Figure S1).

The majority of the cytokines (>70%) and half the growth factors were found in higher levels in the EVs of both patient groups BT compared to their levels in EVs obtained from HC. About 70% of the screened cytokines were decreased by half during treatment in both groups. The most significant reduction, where 90% of the cytokines were reduced by half, was measured in the EVs at the end of the treatment in the combined treatment group.

While 17 and 14% of the growth factors significantly increased during and at the end of the standard treatment, only a slight increase was found in growth factors content in EVs obtained during and at the end of the combination treatment (3 and 0%). Moreover, several proteins were found to be increased only in the standard treatment group (CC-C) and decreased during the combined treatment (CC-W). These include several growth [Activin A, Angiogenin, Angiopoietin-2 (ANG-2), Angiopoietin-like factor IV (ANGPTL4), hepatocyte growth factor (HGF), placenta growth factor (PIGF), Figure 6C], and some inflammatory cytokines [monocyte chemoattractant protein 1 (MCP-1), RANTES (Regulated on Activation, Normal T Cell Expressed and Secreted), the interleukins (IL-6, IL-17) and tissue inhibitors of metalloproteinases (TIMP-1 and 2) (Figure 6D). In contrast, the anti-inflammatory cytokines, such as IL-2 and TIe-2 (the angiopoietin receptor), were increased only during the combined treatment.

## Discussion

The current study analyzed the effects of chemotherapy combined with the support of daily consumption of WGJ on EVs repertoire with the intention to identify reliable biomarkers that would reflect the efficacy of the treatment at the inter-cellular level.

The influence of nutritional support during chemotherapy is a complex research area. For clinical conclusions, studies with large number of patients are needed in order to show small differences in outcome. The current study, using the tool of the EVs, allowed us to reflect the body response to a nutritional supplement, such as WGJ, and may contribute to the understanding of positive influences on body response to chemotherapy that can be changed by a simple, non-toxic nutritional support.

According to previous reports, differential centrifugations cannot provide “pure separation” between exosomes and MVs (20, 21). Therefore, in the current study, we elected to validate total EVs population as they appear in PPP. The results of this study emphasize significant changes between the characteristics of total EVs obtained from different patient subgroups during chemotherapy and the effect of the treatment on EV biogenesis. While no differences were found between the study cohort in EVs size and concentration, we found reduced levels of the exosome markers (CD81 and CD63) in the CC-W group which may indicate that the effect of the WGJ treatment on cell metabolism and function includes EVs biogenesis.

Despite the fact that all patients were adjuvant patients after surgery, some presented very high levels of EVs expressing tumor markers, such as CD66 or A33, before and at the end of treatment, which may indicate the existence of new tumor cells. Median follow-up for both groups at 16 months showed that five patients had died, one in the WGJ group (due to chemotherapy toxicity) and four in the CC-C group (one due to chemotherapy toxicity, two for other reasons, and one due to metastatic disease) (*p* = 0.142). Regarding recurrent disease, four patients in the WGJ group and two in the CC-C group had recurrent metastatic disease. However, the study was not planned to give clinical answers for the use of WGJ as part of the adjuvant treatment of colon cancer patients; such a study would need to include a few hundred patients.

### EVs as a Marker for Endothelial Impairment and Thrombogenicity

In the current study, EVs reflect differences in the vascular state and endothelial impairment between two patient subgroups. Patients who received standard chemotherapy alone presented an increase in endothelial EVs that bear CD144 and CD31^+^/CD41 and probably represent vascular damage (22). The addition of daily WGJ might reduce this effect, as reflected by a decrease in endothelial EVs at the end of treatment. Chemotherapy is known to induce vascular injury and it has been demonstrated that 5-fluorouracil-based therapy induces endovascular injury (23); oxaliplatin is also known as an inducer of hepatic vascular injury (24).

Currently, there are no direct validating measures to correlate chemotherapy treatment with vascular damage or the positive effects of adding WGJ. In the current study, by using unique biomarkers, such as EVs, we were able to monitor the toxic effect of chemotherapy on vasculature and determine the protective and healing effects of the combination of WGJ with chemotherapy on endothelial cells. The reduction of chemotherapy-induced endothelial damage may relate to the effect of WGJ on oxidative stress (25).

The risk for cancer-associated thrombosis (CAT) is dependent on the cancer stage and may be elevated following chemotherapy (26). There is growing evidence indicating that TF-bearing EVs are involved in this process (27, 28). In the current study, the majorities of patient EVs BT were found to be more thrombogenic than the EVs of HC and expressed higher levels of TF, the coagulation initiator protein, and EPCR. The circulating form of EPCR can bind to activated protein C and reduce its availability to deactivate factor Va and VIIIa in the coagulation cascade; therefore, it is considered as a pro-coagulant (29). Negatively charged phospholipids are considered as the first step of apoptosis and also are crucial for coagulation cascade activation. However, they are also an essential step for vesicles blabbing (30). Annexin V is not a coagulation factor, yet its binding indicates the existence of negatively charged phospholipids essential for clot formation and provides a catalytic phospholipid surface on which the “tenase” complex (factor IXa-factor VIIIa) and the “prothrombinase” complex (factor Xa-factor Va) can be assembled. Previously we demonstrated that a percentage of EVs bearing negatively charged phospholipids, labeled by Annexin V, correlated with platelet EVs (31). In the current study, the majority of patients expressed reduced levels of these EVs BT compared to HC, assuming that a decrease in platelet EVs accompanied by degrees of Annexin V labeling EVs are not related to WGJ anti-apoptotic effects.

The EVs thrombogenic character was mainly exhibited in the increase of the TF expression on EVs of patients with standard treatment, while the combined treatment restricted these phenomena (lower levels of TF bearing EVs, TF/TFPI ratios), and reduced expression of negatively charged phospholipids which are essential for the activation of coagulation cascade.

Our recent study demonstrated the potential use of EVs thrombogenicity as a marker for a patient’s hypercoagulable state and its ability to reflect the thrombogenic effects of chemotherapy treatment (15). Specifically, EVs obtained from neo-adjuvant and adjuvant breast cancer patients revealed a higher thrombogenic capacity of EVs drawn at the end of chemotherapy compared to those obtained prior to any treatment. Furthermore, a significant increase in the EV TF/TFPI ratio was found after the first 24 h of doxorubicin treatment. In addition, EVs derived from MDA-MB-231 cells stimulated with high-dose doxorubicin demonstrated significantly increased procoagulant activity compared to EVs isolated from MDA-MB-231 cells pre-exposed to serum-free medium (19).

Overall, the current study demonstrated that, while standard chemotherapy increased EVs procoagulant activity in 40% of patients, combining the treatment with WGJ restrained this effect and increased the procoagulant activity in only 20% of the patients.

### EVs Cytokine and Growth Factors Content

Cytokine and growth factors content can exist in the bloodstream as a free complex or incorporated into EVs. However, it is still debatable if EVs contain significant amounts of proteins and provide a better source of cytokines than whole plasma. The current study presents clarifications in this subject. The ratio (≤1) of protein content as free in plasma (FP) or packed on EVs (Supplementary Figure S1) found in the current study means that the majority of proteins/cytokine were found in higher or equal volume inside EVs than as FP. Encapsulation of cytokine/growth factors in the shield of a phospholipid membrane as happened, for example, with IL1ß (32) has important biological effects, protecting from rapid degradation by proteases, increasing their lasting and promoting penetration to recipient cells by several mechanisms in distant locations.

Angiogenesis mediated by VEGFR plays a central role in tumor proliferation and metastasis, supporting survival and growth of colon carcinoma (33).

In the current study, patients EVs express higher levels of growth factor receptors than healthy persons. Moreover, we found significant differences in EVs VEGFR1, growth factors and cytokine content between the patient subgroups. EVs obtained from the standard chemotherapy treatment group presented an increase in several growth factors content, all related to colon cancer, while lower levels of VEGFR1 labeled EVs, as well as a significant reduction (>50%) in EVs growth factors content during and at the end of chemotherapy were found in the CC-W group. This implies a possible advantage of the combined treatment compared to the CC-C group in terms of disease progression. A previous study demonstrated a link between these growth factors and colon cancer. For example, Activin-A plays a role in the progression of CC and is overexpressed in advanced stages of CC (34). Increasing Angiopoietin-2 and its receptor Tie-2 induces early induction of pro-inflammatory factors related to the development of colon cancer (35), and an increase in angiogenin was found to be correlated with tumor vascularization (36). An increase in inflammatory cytokines is related to the development of colon cancer (37). The anti-inflammatory effect of WGJ was demonstrated in the reduction of EVs content of several cytokines, all of them involved in cancer progression. MCP-1 induces recruitment of macrophages to the tumor microenvironment, RANTES, involved in colon cancer progression (38, 39), plasma levels of TIMP-1 found to be markers of primary CC (40), IL-6 regulates chronic inflammation and promotes tumor cell proliferation and survival (41), and IL-17 induces PD-L1 protein expression in human colon cancer cells (42).

The anti-inflammatory effect of WGJ, as reflected in patients EVs, may explain two small clinical studies (43). The first study showed positive results in 15 patients with rheumatoid arthritis (RA), following a one year intake of WGJ as supplementation to standard therapy, resulting in improved symptoms in all patients who had failed at least two disease-modifying anti-rheumatic drugs previously. In the second study, a small randomized trial with ulcerative colitis patients has shown greater improvements in overall symptoms and in the severity of rectal bleeding compared to patients who received a placebo, which was similar in appearance but not in taste or smell (44). Overall, the differences in cytokines and growth factors levels may explain the anti-cancer activity documented in an animal model of colon tumors established in rats injected with the carcinogen azoxymethane and which were greatly decreased following the administration of WGJ (45).

## Study Limitations

Research based on choice rather than a randomized trial may result in patient-related bias (volunteer bias). However, the same characteristics that motivated patients in the study sample to choose wheatgrass consumption most likely resemble the characteristics that would motivate patients in the general population to consume wheatgrass (e.g., tendency toward healthy diet, health habits). In addition, while HC is mostly females, the patient groups (CC-C and CC-W) are mostly men, which may lead to gender bias.

Despite the fact that no significant differences were observed between baseline demographic and clinical characteristics of patients treated with WGJ vs. patients treated with standard treatment only, some differences between the two subgroups were found in the patients EVs BT. We cannot say if this is an artifact of the experiment or patients EVs of the combined treatment group. If so, we can only speculate that there are other unknown parameters that influence patients EVs content. To overcome this discrepancy and naturalize the effect of the differences between EVs at the first time point of measurement, in some parameters we calculated the change in EVs characteristics during and at the end of treatment compared to their levels BT in each patient subgroup.

The study cohort is relatively small and the chemotherapy regimen is different among the 50 patients. The sub-group of patients treated only with capecitabine was too small to show differences that may exist in EVs characterization related to the use of oxaliplatin. It may be that enlarging the study group will reach higher statistical significance; therefore, additional studies need to be done. The EVs characterization was based mainly on total EVs population that appeared in PPP without further fractionation. The EVs are a mixture of large and small vesicles. Further research shall be performed in order to identify the effects of different EVs populations (e.g., large vs. small).

## Conclusion

EVs of colon cancer patients treated with chemotherapy combined with WGJ showed lower levels of thrombogenicity, endothelial markers and inflammation proteins compared to chemotherapy treatment only. Differences in these factors, related to wheatgrass consumption, may indicate improvement of patients’ pathophysiology and EVs may serve as biomarkers for treatment efficacy.

## Data Availability Statement

All datasets generated for this study are included in the article/Supplementary Material.

## Ethics Statement

The study was approved by the Institutional Review Board of Rambam Health Care Campus (RHHC) in Haifa, Israel (Approval No. 0153-13). The patients/participants provided their written informed consent to participate in this study.

## Author Contributions

AAh and GB-S: methodology. AAv, AAh, and GB-S: validation, formal analysis, investigation, data curation, and writing – original draft preparation. AAv, AAh, GB-S, and TB: resources. AAh, GB-S, and TB: writing – review and editing. BB, MC, and MM: supervision. All authors contributed to the article and approved the submitted version.

## Conflict of Interest

The authors declare that the research was conducted in the absence of any commercial or financial relationships that could be construed as a potential conflict of interest. The reviewer EB-A declared a past co-authorship with one of the authors GB-S to the handling editor.

## Supplementary Material

FIGURE S1 Ratios of protein content free in plasma or packed in EVs in the study cohort. EV proteins extract was obtained from a pool of five specimens within each patient subgroup were validated by Human Angiogenesis Protein Antibody Array. Ratio of protein content as free in plasma (FP) or packed on EVs in the study cohort. **(a)** FP-HC/EVs-HC; **(b)** FP-CC-C BT/EVs-CC BT; **(c)** FP-CC-C3/EVs-CC3; **(d)** FP-CC-W BT/EVs-CC-W BT; **(e)** FP-CC-W3/EVs-CC-W3.
