# Supplementary figures and images for: Extracellular Vesicles Reflect the Efficacy of Wheatgrass Juice Supplement in Colon Cancer Patients During Adjuvant Chemotherapy
Source: Front Oncol. 2020 Aug 26;10:1659. doi: 10.3389/fonc.2020.01659 (PMC7479215; doi:10.3389/fonc.2020.01659)

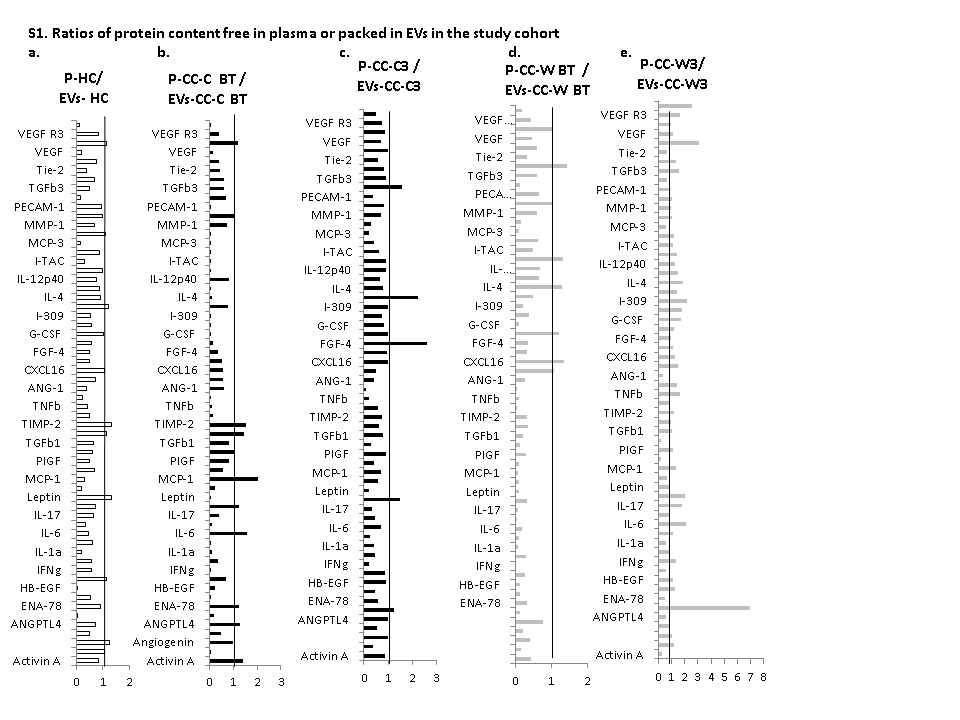

Supplement: FIGURE S1 — Ratios of protein content free in plasma or packed in EVs in the study cohort. EV proteins extract was obtained from a pool of five specimens within each patient subgroup were validated by Human Angiogenesis Protein Antibody Array. Ratio of protein content as free in plasma (FP) or packed on EVs in the study cohort. (a) FP-HC/EVs-HC; (b) FP-CC-C BT/EVs-CC BT; (c) FP-CC-C3/EVs-CC3; (d) FP-CC-W BT/EVs-CC-W BT; (e) FP-CC-W3/EVs-CC-W3. [file Image_1.TIF]
